# A comparative genomics approach to identifying the plasticity transcriptome

**DOI:** 10.1186/1471-2202-8-20

**Published:** 2007-03-13

**Authors:** Andreas R Pfenning, Russell Schwartz, Alison L Barth

**Affiliations:** 1 Department of Computer Science, Carnegie Mellon University, Pittsburgh, PA 15213, USA; 2 Department of Biological Sciences, Carnegie Mellon University, Pittsburgh, PA 15213, USA; 3 Center for the Neural Basis of Cognition, Carnegie Mellon University, Pittsburgh, PA 15213, USA

## Abstract

**Background:**

Neuronal activity regulates gene expression to control learning and memory, homeostasis of neuronal function, and pathological disease states such as epilepsy. A great deal of experimental evidence supports the involvement of two particular transcription factors in shaping the genomic response to neuronal activity and mediating plasticity: CREB and zif268 (egr-1, krox24, NGFI-A). The gene targets of these two transcription factors are of considerable interest, since they may help develop hypotheses about how neural activity is coupled to changes in neural function.

**Results:**

We have developed a computational approach for identifying binding sites for these transcription factors within the promoter regions of annotated genes in the mouse, rat, and human genomes. By combining a robust search algorithm to identify discrete binding sites, a comparison of targets across species, and an analysis of binding site locations within promoter regions, we have defined a group of candidate genes that are strong CREB- or zif268 targets and are thus regulated by neural activity. Our analysis revealed that CREB and zif268 share a disproportionate number of targets in common and that these common targets are dominated by transcription factors.

**Conclusion:**

These observations may enable a more detailed understanding of the regulatory networks that are induced by neural activity and contribute to the plasticity transcriptome. The target genes identified in this study will be a valuable resource for investigators who hope to define the functions of specific genes that underlie activity-dependent changes in neuronal properties.

## Background

Transcription of new genes is initiated in the nervous system by both synaptic activity and action potential firing [1-3], and activity-dependent changes in gene expression are critical in epileptogenesis, brain injury, and learning and memory. For example, reducing CREB-dependent gene expression eliminate memory acquisition [4], and knock-out of the gene encoding zif268 (egr-1, krox24, NGFI-A) impairs memory-like processes such as LTP [5]. Alterations in gene expression after seizure may lead to abnormal neural function and the development of epilepsy (reviewed by [6,7]). Furthermore, a number of inherited forms of mental retardation can be traced to defects in activity-dependent gene expression, such as Rubenstein-Taybi syndrome, related to a mutation in the CREB-binding protein, and Rett syndrome, tied to a defect in a DNA-binding protein that regulates the correct timing of expression of many downstream genes [8]. Although some genes that are rapidly upregulated by neuronal activity have been identified, the direct and indirect targets of activity-dependent transcription factors remain of substantial interest in developing and constraining models for how neuronal function is altered by experience.

Various prior approaches have attempted to identify gene sets that underlie activity-dependent changes in neural function. cDNA microarrays have been frequently employed to characterize the plasticity transcriptome by identifying candidate genes that are upregulated after activity [9-15], typically through pharmacologically-induced seizure [16-21] or patterned sensory stimulation [22,23]. Alternate approaches include the use of chromatin-immunoprecipitation (ChIP) against specific transcription factors and determination of immunoprecipitated DNA binding sites by hybridization to microarrays or PCR analysis [24-28]. Both approaches, though informative, have important limitations. For example, current cDNA microarray analyses have typically identified genes that are show several-fold regulation (>1.5 up- or down-regulation), although in principle microarray studies can identify genes showing small changes in transcript levels given adequate numbers of redundant measurements. Differences in the timing and conditions of sample collection for microarray analysis can lead to heterogeneous results between investigators. In addition, microarray analysis typically does not reveal information about the transcriptional pathways by which genes are regulated in an activity-dependent manner. Although ChIP has been a useful approach for identifying candidate transcription factor binding sites, interpretation of this data can be complicated by tissue-specific occupancy of binding sites and the inherent heterogeneity of cell types in brain tissue [29]. For example, ChIP cannot identify occupied sites if they occur in only a small subset of neurons. An alternate approach to identifying potential activity-regulated genes has been to isolate specific candidates and examine changes in the abundance of their protein or mRNA after manipulations of activity (see for example, [30,31]). However, this approach is obviously limited because it must proceed in a highly directed, case-by-case manner, and does not accommodate the discovery of novel or unlikely gene candidates.

Identification of activity-regulated genes could be improved by genomic screens that are unbiased by cell type, target gene preselection, or average expression level. A growing body of knowledge regarding transcriptional regulation of gene expression and consensus sequences for transcription factor binding, combined with annotated genome sequences, makes it possible to carry out a directed search for binding sites of specific activity-regulated transcription factors at the scale of an entire genome. Methods for locating transcription factor binding sites often rely upon relatively simple comparisons of a single sequences or consensus binding sites with individual promoter regions [32-34]. Although such searches can be productive, the use of a single consensus site is problematic because every nucleotide must be analyzed independently from the rest of the binding site and degeneracy can easily be over- or underestimated. This results in subjective target identification whose reliability is difficult to judge.

Here we develop and employ a rigorous computational method for scanning gene promoters for binding sites of two known activity-dependent transcription factors: CREB and zif268. These transcription factors were selected based upon a large body of data that links their activity to adaptive responses to neuronal activity, especially activity occurring during learning [35]. For example, transduction of extracellular signals leads to CREB phosphorylation and assembly of the transcriptional apparatus, events that are necessary for consolidation of long-term memory [36]. Overexpression of a constitutively active form of CREB enhances LTP in mice [37], and genetic manipulations that reduce CREB transcriptional activation result in impaired learning and memory [4,38]. Zif268 is an immediate-early gene (IEG), whose expression can be upregulated following increased neural activity and activation of intracellular signaling cascades [39] and is required for some forms of learning [5,40]. Because activity-dependent changes in gene transcription are linked to memory consolidation and also occur as a response to pathological conditions such as seizure [41-43], identification of the downstream targets of these transcription factors remains of considerable interest.

We performed an *in silico *analysis for full CREB and zif268 binding sites of promoter regions from all available annotated mouse genes. Likely binding sites were identified by applying position-specific scoring matrices (PSSMs) derived from previously characterized binding sites for CREB1 and zif268 confirmed by *in vitro *assays [44,45]. Individual binding site examples from specific genes were not used to develop the scoring matrix to provide consistency to the method used to develop the training algorithm without biasing it to a few anecdotal examples from individual genes. Because this analysis was specific for longer consensus binding sequences (i.e. not half-sites), it is more stringent and has a lower false positive rate than previous searches [46].

CREB sites have been demonstrated near both coding and non-coding regions of the genome and there is a growing body of data that supports an important regulatory role for CREB, and possibly other activity-dependent transcription factors, in non-coding regions (specifically for regulatory, noncoding mRNAs; [47]). Nonetheless, we restricted our search to promoter regions of annotated genes because locations of non-coding RNAs are currently not adequately annotated and genome-wide scans necessitate more stringent statistical tests than limited scans of promoters, and would result in a substantial increase in false negative rates.

As a further test of the validity of these transcription factor binding sites, we performed this search in parallel on all human as well as rat genes with annotated transcription start sites. In general, because the rat genome is less well-annotated that either mouse or human, most analysis was carried out on mouse and human candidate genes. Dual hits from both the mouse and human genomes were considered more likely to have an activity-dependent component to their regulation. Our results identify 516 candidate genes with conserved CREB or zif268 binding sites in both mouse and human homologous genes that may be regulated by activity, six of which were predicted to have more than one type of transcription factor binding site. These results provide an important resource in understanding the regulatory networks that control activity-dependent programs of gene expression.

## Results

We used a comparative genomics approach to identify genes likely to be regulated by neural activity. Position-specific scoring matrices were employed to create a search algorithm for DNA binding sites for CREB and zif268 in the promoters of annotated genes in order to define candidate genes from the plasticity transcriptome to guide and constrain future analysis. Gene lists were refined and evaluated by comparison of target frequencies across species and the presence of specific transcription factor binding sites in conserved homologous genes between human and mouse genomes. These gene lists allowed us to identify a significant subset of target genes that may be regulated in common by CREB and zif268. Finally, special attention was paid to the presence and relative frequency of genes with specific neural relevance amongst the candidate dataset, with an eye toward future experimental focus on the activity-dependant regulation of these genes.

### Developing binding site consensus sequences

**Figure 1:**
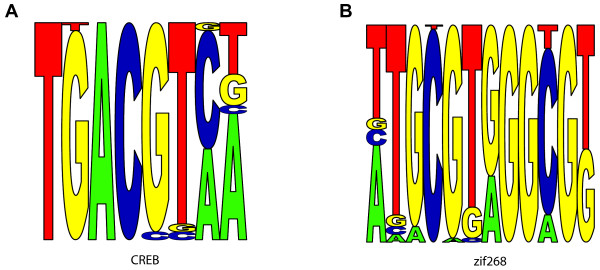
Activity dependent transcription factor binding sites consensus sequences. The height of the letters is proportional to their frequency in the data used to build the matrix. For instance, a large "A" in position one means that "A" belongs to the most probable consensus for that transcription factor binding site. (A) Consensus sequence given by the transfac CRE-binding matrix V$CREB_01. (B) Consensus sequence given by the transfac zif268 binding matrix V$EGR1_01.

Previous analyses of transcription factor binding sites have suffered from low degeneracy based upon comparison of a target sequence to a single overrepresented binding site or high degeneracy and a high false positive rate based upon inclusion of related IEG subfamily members with similar but distinct sequence specificities. When related transcription factors (such as zif268, egr-2, and egr-3 or c-fos/c-jun and FosB/JunD heterodimers) with overlapping binding site consensus sequences are pooled, binding site consensus is significantly relaxed and false positive rates are increased. Our method sought to reduce the rate of false positives by using a smaller number of experimentally verified, high-quality transcription factor binding sites using frequency matrices that have been experimentally developed for zif268 [45,48] and CREB [44,48]. A schematic of the consensus sequences used is shown in Fig. 1.

An analogous search was attempted with AP-1, whose components, fos and jun, are also IEGs. The resulting predicted targets were not enriched for promoter regions and showed little conservation across species, facts we attribute to excessive degeneracy of the known AP-1 binding sequences used in training the computational methods. While we believe this negative result helps to reinforce the significance of the positive CREB and zif268 results, we have omitted further discussion of AP-1 in this report.

### Computational genomic analysis: mouse, rat, and human genomes

**Figure 2:**
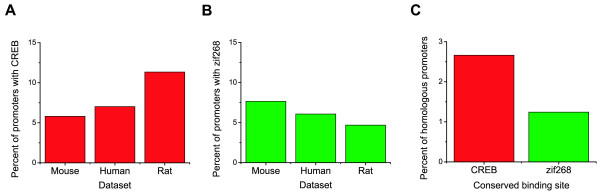
Relative frequency of transcription factor binding sites within promoter regions across species. (A) The percentage of human, mouse, and rat promoters with a predicted CREB binding site. (B) The same as (A), but for zif268. (C) The percent of genes within the homologene dataset showing a CREB or zif268 site, calculated by (# of promoters with a binding site × 100)/(# of human and mouse homologous pairs).

In order to identify conserved gene candidates subject to activity-dependent regulation, the entire database of mouse, rat, and human promoter sequences was searched for CREB and zif268 binding sites. In total, 18,071 mouse genes, 5,943 rat genes, and 19,794 human genes were examined (summarized in Table 1; full gene lists can be found in Additional Files 1, 2, 3, 4). This represents all the annotated promoters from these species (approximately 50% of the estimated distinct mouse coding regions and two-thirds of the total estimated distinct human genes; [49-51]). CREB binding sites were predicted in 6% of mouse promoters, 7% of human promoters, and 11% of rat promoters (Fig. 2a). Zif268 binding sites were predicted in 8% of mouse promoters, 6% of human promoters and 4.6% of rat promoters (Fig. 2b). The precise sequence and location of binding site for each species is annotated in Additional Files 5, 6, 7. Because the mouse and human gene sets were more complete and representative of the total number of coding regions within those species than found in the rat dataset, we will primarily refer to the human and mouse searches from this point onward.

As we were interested in using comparative genomics to improve hit quality, we also examined those genes with homologues in the mouse and human datasets, a total of 13,365 genes (homologene dataset; [52]). In general, the frequencies of binding sites predicted for genes in the homologene dataset for either species in isolation were similar to those found for non-homologous genes in the same genomes (Table 1), suggesting that the homologous genes are a representative set of genes with respect to binding site predictions. We then examined the frequencies of binding site predictions conserved across both members of a gene pair. The number of hits from this search was lower than observed for individual genomes: in the set of mouse-human homologues, 2.66% have a conserved CREB site and 1.24% have a conserved zif268 site (Fig. 2c).

### Estimating rate of false positives

To develop a rigorous estimate of the quality of identified targets, we applied two methods for calculating their positive predictive value, defined as the probability a predicted hit is correct (see Methods for a complete description). This measure provides a conservative estimate of the percentage of identified transcription factor binding sites that we expect to be functional binding sites. For this purpose, we examined binding site location and cross-species conservation.

**Figure 3:**
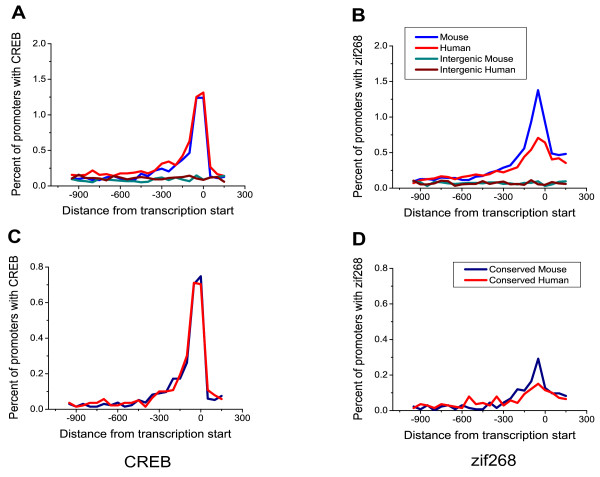
CREB and zif268 binding sites show strong location specificity. All histograms were created using a bin size of 50 bp. The total number of binding sites in each 50 bp region was divided by the total number of promoters for that dataset. Shown are both human (red) and mouse (blue) and promoters. For the intergenic dataset, the area shown is from -51,200 bp to -50,000 bp relative to transcription start. The mouse dataset is the location of mouse binding sites when the homologous gene also has a binding site of the same type. The human dataset is the location of human binding sites when the homologous gene also has a binding site of the same type. A) Promoters with a CREB binding site are grouped by position relative to transcription start, showing pronounced location specificity within the promoter. (B) The same as (A) but for zif268. (C) The same as (A) but for the conserved CREB targets in the mouse-human homogene dataset. (D) The same as (A) but for the conserved zif268 targets in the mouse-human homogene dataset. The percentage of promoters with a binding site is calculated relative to the total number of homologous pairs.

Fig. 3 shows an analysis of position specificity of binding site predictions within the promoter regions. A pronounced peak in the relative frequency of binding site locations was observed for both CREB (Fig. 3a; see also [46]) and zif268 (Fig. 3b). Both binding sites were more than five times more likely to occur in the 50 bp closest to the annotated start site compared to more distant or intergenic sequence. This frequency increase was observed in analysis of the mouse and human gene datasets, as well as in the conserved mouse and conserved human gene datasets (Fig. 3c and 3d). Furthermore, the low frequency of binding sites in regions most distal to the annotated start site (i.e. 800 bp upstream) was comparable to the frequency in intergenic regions (see Methods), suggesting that our selection of a 1,000 bp region surrounding transcription start was sufficient to capture most of the meaningful binding sites that might regulate gene expression. Using intergenic (i.e. 50,000 nt upstream of the annotated start site) hit frequencies to estimate false positive rate, we derived a positive predictive value of 0.64 for CREB hits in human and mouse promoters. Zif268 had a slightly higher positive predictive value of 0.81 in mouse and 0.74 in human (Table 1). The full set of predicted binding site locations is provided in Additional Files 1, 2, 3, which present the gene, location relative to transcription start, and sequence of each putative binding site for the mouse, human, and rat genomes, respectively.

Comparative genomics was used to provide a second method of estimating the positive predictive value. In the set of mouse-human homologues, 2.7% have a conserved CREB site and 1.2% have a conserved zif268 site. We proposed that the set of homologue pairs with conserved binding sites would provide a conservative set of true positive hits. The positive predictive value of the hits could then be estimated from the excess of conserved hits beyond what would be predicted from the hit rates in the individual genomes on the assumption of independence between genomes (see Methods). In contrast to the results using location specificity, the conservation approach yielded higher estimates of positive predictive value for CREB (0.83) than for zif268 (0.56). This difference is most likely to due changes in zif268 targets or promoter GC content between species. We can further validate these results by measuring conservation in binding site positions for the conserved hits. Comparing the positions of the conserved predicted sites between mouse and human yields correlation coefficients of 0.18 for CREB (p-value < 0.001) and 0.21 for zif268 (p-value < 0.01). There is considerable noise in location specificity, which might be explained by insertions or deletions in the promoters since the separation of human and mouse lineages or by errors in annotated transcription start sites in either species. The positions nonetheless show significantly greater correspondence than can be explained by chance.

### Overlap of CREB and zif268 target genes

Prior analyses of activity-dependent gene expression have not addressed whether the transcription factors selected for analysis regulate separate or partially overlapping target genes. We reasoned that genes containing binding sites for both transcription factors might be more likely to have an essential role in mediating activity-dependent changes in gene expression. To examine this, we looked at the relative frequencies of genes with both CREB and zif268 binding sites in the mouse, human, and rat gene datasets compared to the frequencies of genes with either site in isolation. Target genes were generally non-overlapping, suggesting that each transcription factor may regulate a distinct subset of genes, a possibility that might allow a cell greater combinatorial control over stimulus-specific transcriptional programs.

Intriguingly, however, the amount of overlap between zif268 and CREB targets was greater than would be expected by chance, suggesting that at least a subset of target genes may be coregulated by the two transcription factors analyzed. Statistical analysis showed that the predicted binding sites shared more targets in common than would be expected by chance for both mouse and human genomes, with the results weakly significant for mouse (*p *< 0.05), but not significant for human (*p *= 0.18).

**Figure 4:**
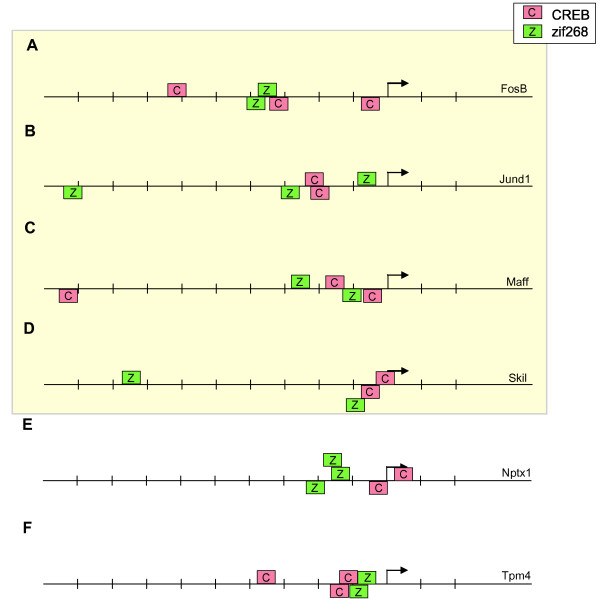
Binding site location in CREB/zif268 double hits. Promoter regions for the genes on the line to the left are from -1000 bp to 200 bp relative to transcription start, which is denoted by the arrow. The red blocks above represent CREB binding sites while the green blocks represent zif268 binding sites. Mouse transcription factor binding sites are found on top of the line while human sites are below. A non-alignment method was used to identify promoter regions, so homologous binding sites might not be at the same location. Gene symbols shown are those for mouse. Promoter regions shown are for (A) *FBJ osteosarcoma oncogene B/FosB*, (B) *Jun proto-oncogene related gene d1 *(C) *v-maf musculoaponeurotic fibrosarcoma oncogene family, protein F (avian)*, (D) *SKI-like*, (E) *neuronal pentraxin 1*, and (F) *tropomyosin 4*. The targets in the yellow box represent transcription factors.

Only six genes with conserved binding sites for both transcription factors were found. A significant fraction of these common targets were transcription factors. FosB (Fig. 4a), Jund1 (Fig. 4b), and Maff (Fig. 4c) are all members of the AP-1 family of transcription factors. The Skil (Fig. 4d) transcription factor is a member of the SKI/SNO/DAC family which are known to associate with AP-1 under some conditions [53]. The observation that a specific group of transcription factors can be regulated by both CREB and zif268 implicate these genes in transcriptional networks of activity-regulated gene expression.

### Computational analysis of target gene sets

We sought to characterize the functional properties of the derived gene set through an unbiased computational search for functional gene classes significantly over- or under-represented in our hit set. We conducted this analysis by applying the GOstat web resource [54] to the set of CREB and zif268 conserved and species-specific targets. Because there were relatively few conserved targets in the homologene datset, GOstat analysis found few hits, such as RNA processing and localization, only for CREB target genes.

Because of the increased statistical power of using more data (versus higher quality data), we chose to further this analysis using the species-specific gene target lists. We chose to present the mouse target list because the comparative data available has primarily been carried out in rodents. As with the conserved gene list, genes with CREB consensus sites showed significant overrepresentation for targets involved in RNA processing, but were underrepresented in electrophysiologically important transmembrane receptor targets (Additional File 8). However, the few receptor and channel targets identified in this analysis may have critical functional importance. For example, seizure-dependent changes in expression of three targets, Kcnk1, Kcnmb4 and Hcn2, have been found [55], and these two genes represent targets for neuroprotective or anticonvulsant agents. Targets of zif268 are also underrepresented for transmembrane receptors, but are overrepresented for transcription factors as well as genes with neural-specific functions such as neuron development and axonogenesis. A full list of these targets can be found in Additional File 9.

### Comparison to experimental analyses

Many of the gene targets identified in this analysis have been identified in previous studies. Published experimental data for the number of putative CREB and zif268 targets suggests that there may be hundreds of specific genes that carry these binding sites (see for example, [56,57], but much of this data is indirect. We thus surveyed the literature for examples of CREB and zif268 target regulation, using direct evidence for CREB or zif268 binding or consensus sequence identification. A comparison of some prominent candidates from this study with a literature-derived set of known CREB and zif268 targets is shown in Table 2. Strong experimental support for many genes that were identified in the present analysis was found, such as somatostatin [58], tyrosine hydroxylase [59,60], and synapsin II [61]. Because target regulation has been shown to depend critically on brain area, developmental stage, and even strain differences between mice and negative results are thus uniformative, we did not choose to select a random subset of genes to experimentally pursue as part of this purely computational analysis.

Although there are few well-documented differences between the transcriptional regulation of homologous genes in mouse and humans, our analysis was successful at identifying one of the few well-characterized mutations in a CREB binding site between a mouse and human gene that influence transcription, the glycoprotein hormone alpha subunit [62]. This gene carries an identified CREB site in human, and a single gene mutation in the CREB binding site in mouse abolishes placental expression of this gene. Accordingly, this gene was identified as a human but not a mouse CREB target gene.

Several experimental studies have sought to characterize CREB or zif268 targets by searching for genes upregulated after transcription factor activation. We compare the genes isolated in a subset of these studies to the targets identified here. Microarray results of regulated genes after 1–5 weeks of hippocampal overexpression of a constitutively active form of CREB [37] were compared to the results from our search, using a program that takes into account relative changes in transcript levels [63]. We found that predicted CREB targets from our search were overrepresented in that microarray dataset, but by a statistically insignificant amount. An analysis of CREB targets from a similar computational search [64]showed slightly less enrichment in this microarray dataset, but this enrichment was also not significant. The lack of significance in overlap is likely due to the extended duration of VP-16 CREB overexpression, which complicates an interpretation of this result, as well as the fact that we searched specifically for direct targets of CREB while a microarray study would be expected to capture both direct and downstream targets.

Comparison of our work to the results of another experimental study that used chromatin immunoprecipitation followed by PCR amplification of CREB-associated DNA [25] revealed minimal overlap of genes (Table 3). This lack of overlap may in part be due to tissue specific CREB-site occupancy [29] and the small number of target genes compared. It may also derive from the fact that differences in annotation made exhaustive comparison of the two gene sets difficult; rather our analysis was based on a non-exhaustive set of results reported in the body of the paper by Impey et al. [25]. It is notable that even the study by Impey et al. failed to identify some well-established CREB target genes, such as somatostatin, indicating that neither our computational nor their ChIP-based gene lists are likely to be exhaustive.

We also compared overlap between zif268 targets identified in our computational analysis and a recent zif268 overexpression study [49]. We observed an extremely low rate of overlap between these two studies, where only 6.7% (9/135) regulated genes were represented in either the mouse or human zif268 target lists generated in our study (Table 3). Only one gene, the small glutamine-rich tetratricopeptide, showed a conserved zif268 site in both mouse and human as well as regulated mRNA levels after zif268 overexpression. The relatively low level of target overlap in this and other studies may be due to the duration of transcription factor overexpression (days) and ensuing cascades of altered gene expression (i.e., not all targets are directly regulated by the overexpressed transcription factor). Our decision to favor a high-specificity gene list at the cost of lower sensitivity was likely also a factor. While the exact criterion used by James et al. study [57] to define target sites was unclear, likely differences in the target specificity/sensitivity trade-off would be expected to yield poor correspondence between the data sets.

### Comparison to other computational analysis

A similar comparative genomics analysis was carried out by Conkright et al., using a hidden Markov search algorithm trained on ten well-characterized CREB binding sites [64]. Of 78 conserved CREB targets identified by Conkright et al. and 356 identified by our study, 25 were common to both studies (Table 2). Although this is a strong overlap relative to what one would expect by chance, it is nonetheless curious that it was not higher. We attribute this fact to likely assignment errors in both sets as well as likely differences in annotation. We believe our study is likely to have yielded a higher quality set of predicted binding sites based on the fact that we have access to more recent genome annotations, search in a more tightly focused region (1.2 kb versus 10 kb), search relative to transcription rather than translation start, and use a prediction algorithm that would screen out some possible spurious predictions likely with a hidden Markov model approach. This methodological argument that our gene set is closer to the ground truth is supported by the fact that while the two studies predict comparable numbers of CREB sites in mouse and human individually (1050 mouse and 1389 human for the present study and 1349 mouse and 1663 human for Conkright et al.) our predicted sites were validated by cross-species conservation at a rate several-fold higher (356 validated versus 78). The similarities of our gene set to that of Conkright et al. thus provide good validation that both approaches find meaningful gene sets, but the deviations do not challenge the accuracy of our set.

## Discussion

We have applied computational analyses to identify candidate genes regulated by neural activity based upon the presence of CREB and zif268 binding sites within their promoters. This work combined sequence-based motif finding methods with an analysis of homology, binding site co-occurrence, and binding site location to estimate and improve prediction accuracy. Because the consensus sites used for analysis were not derived from a possibly unrepresentative subset of specific genes but rather from experimentally determined binding motifs [44,45], we believe that the gene lists presented here are uniquely unbiased. The generated candidate gene lists provide potential targets for future experimental validation and may also be useful for interpretation of microarray data and inference of gene regulatory networks. This work has also revealed a pronounced location-specificity of high-quality CREB and zif268 binding sites, an observation that may be a diagnostic criterion for the detection of binding sites near poorly-annotated non-coding regions as well.

The principal goal of this work was the generation of a computational resource identifying likely targets of activity-dependent regulation to help guide future experimental study. Unlike previous experimental studies, which identified both direct and indirect targets using microarray analysis of regulated genes following overexpression of activated CREB or zif268 [37,56], this study specifically identified high-quality, direct transcriptional targets of CREB and zif268. Based on our comparative genomic analysis, we believe that our list of predicted targets based on conserved binding site predictions has a very low false positive rate. Although experimental support for some targets was not observed, this is hardly surprising given that site occupancy has been shown to vary according to tissue type [56,65-67] and overexpression of zif268 has been associated with repression of genes carrying zif268 sites within their promoter regions [56]. Omission of real candidate genes may have occurred due to 1) the high stringency of the search we carried out, 2) poor or incomplete annotation of transcription start sites or multiple transcription start sites for a single gene, and 3) real differences in the regulation of mouse and human genes. It is also important to note that actual CREB binding may be influenced by chromatin superstructure as well as the presence of additional regulatory factors, variables that were not examined in the biochemical studies that provided the sequence matrices for CREB and zif268 that we used here. However, several lines of evidence confirm that this approach identifies a gene set that includes expected CREB and zif268 targets, shows activity-dependent expression changes and is significantly enriched for several specific functional classes of proteins consistent with a role in activity-dependent regulation.

The work also suggests several avenues for improvement of computational approaches to identifying targets of transcriptional regulation. One of the most striking findings from this study was the pronounced location specificity to CREB and zif268 targets within annotated gene promoters. The frequency of CREB and zif268 sites was greatest within the 50 bp closest to the annotated start site, and dropped to baseline (i.e. intergenic) frequencies after 600 bp. This finding suggests that the functionality of these sites is greatest at proximal locations within the promoter, a conclusion that is further supported by the high conservation of location specificity between species. Our results strongly support the use of homology methods for improving specificity of binding site prediction [68], as well as the use of binding site co-occurrence for the same purpose [69,70]. Our inability to replicate these successes with AP-1, though, highlights the necessity of a strong set of experimental data in these computational approaches.

Finally, this study has also led to some intriguing findings about the specific likely targets of CREB and zif268. Although these transcription factors are not exclusively expressed in the CNS – indeed, they are present in many other cell types at different developmental stages and regulate the transcription of gene targets that are not specifically neural – the essential role that they play in neural plasticity was an important consideration in motivating this study and the targets identified will be of strong interest to neurobiologists.

Significantly, the study revealed a significant convergence of targets containing both CREB and zif268 binding sites. Among these putative CREB and zif268 co-regulated genes, the set conserved across mouse and human included several known transcription factors and transcription regulatory elements, most associated with AP-1 regulation. CREB and zif268 may represent the top level of a regulatory network implicated in neural function, with expression of the functional proteins primarily controlled by an intervening network of other regulatory factors. Indeed, it is clear from other experimental data as well as our own that additional waves of transcriptional regulation and distinct programs of gene expression will follow the activation of this initial cascade [6]. Furthermore, neural activity may also induce various forms of non-transcriptional regulation, including alternate splicing, accelerated degradation, or altered intracellular trafficking that the present analysis did not address. Thus, the target set presented here is likely only a beginning towards characterizing the full complement of genes induced by neural activity. The problem of defining the plasticity transcriptome is thus likely to remain an exciting challenge for computational and experimental researchers for the foreseeable future.

## Conclusion

Computational identification of putative targets of CREB and zif268 regulation has identified a set of likely direct targets of activity-dependent regulation that avoids biases inherent in current experimental methods for characterizing such sets. In addition to providing a candidate gene set for future analysis, the study has revealed a pronounced location specificity and bias for co-occurrence particularly in promoters of other transcription factors, which will be useful for improving detection algorithms and more completely characterizing the regulatory networks underlying activity-dependent gene expression.

## Methods

### Promoter database

We compiled a database of gene promoter regions using sequences from mouse build mm6 [71], rat build rn3 [72], and human build hg17 [49,51] of the UCSC Genome Bioinformatics Resource [73]. Transcription start sites for these promoters organized by mRNA accession number were found in the table "knownGene.txt" for each build. Where promoter regions were reported within 50 bp of each other, only the one earlier on the chromosome was used, as the copies were presumed to be duplicates of the same promoter region (derived from otherwise identical mRNAs of different lengths). Incomplete promoters with missing sequence data were also removed from the analysis. Annotated promoters included both TATA-box containing and TATA-less genes. The full promoter list was annotated with gene name, symbol, and accession number using the NCBI gene resources [52,74]. In total, 18,071 mouse promoters, 19,794 human promoters, and 5,943 rat promoters were analyzed (Tables 1, Additional files 1, 2, 3).

When searching for candidate genes, we defined a putative promoter to be the genetic sequence from -1,000 bp to +200 bp of each transcription start for human, mouse, and rat genes. A set of intergenic sequences was also compiled for human and mouse to construct a "random" control dataset of 1,200 bp sequences using the regions from -51,200 bp to -50,000 bp relative to each transcription start site, where the transcription factors CREB and zif268 are not likely to have regulatory function. Due to a decrease in sequence quality further away from transcription start, distal sequence regions were available for only 77% of total genes, leaving 13,475 mouse intergenic regions and 15,178 human intergenic regions far analysis (Table 1). In order to confirm location-specificity trends inferred from the 1,200 bp regions, an additional search was run for each gene on an extended promoter region (-6,000 bp to +200 bp). We saw no significant difference in the region between -1,000 bp and -6,000 bp compared to the -51,200 bp to -50,000 bp region, suggesting that the initial search had identified the majority of sites with likely function.

The Homologene database provided us with human and mouse homologous pairs based on gene accession number [52], yielding 13,365 homologous gene pairs (Table 1, Additional File 4). A binding site prediction was defined as conserved if the same binding site type was predicted in the promoters of both homologous genes, without regard for position in the promoter.

### Transcription factor binding site inference

The goal of this search was to identify a transcription factor binding site compared to its background. This is the case when the probability that it is a binding site is greater than the probability that the sequence would be observed by chance.

*P*(*Model*) > *P*(*Background*)

log(*P*(*Model*)) > log(*P*(*Background*))

log(*P*(*Model*)) - log(*P*(*Background*)) > 0

Position specific scoring matrices (PSSMs) or position weight matrices (PWMs), are a well-established method of motif finding [75,76]. We used a variant of them to find the log probability of a sequence being a part of the model. These methods are similar to the transcription factor binding site search available through the database of transcription start sites [77]. Binding site frequency matrices for CREB and zif268 were obtained from the Transfac [48,78] public database (see Fig. 1). These matrices give the frequency of each nucleotide in each position of the binding site. Scoring matrices for the present study were created from the Transfac frequency matrices with the following equation:

Sn,p=log⁡(An,p+b∑iAi,p+4b)
 MathType@MTEF@5@5@+=feaafiart1ev1aaatCvAUfKttLearuWrP9MDH5MBPbIqV92AaeXatLxBI9gBaebbnrfifHhDYfgasaacH8akY=wiFfYdH8Gipec8Eeeu0xXdbba9frFj0=OqFfea0dXdd9vqai=hGuQ8kuc9pgc9s8qqaq=dirpe0xb9q8qiLsFr0=vr0=vr0dc8meaabaqaciaacaGaaeqabaqabeGadaaakeaacqWGtbWudaWgaaWcbaGaemOBa4MaeiilaWIaemiCaahabeaakiabg2da9iGbcYgaSjabc+gaVjabcEgaNjabcIcaOmaalaaabaGaemyqae0aaSbaaSqaaiabd6gaUjabcYcaSiabdchaWbqabaGccqGHRaWkcqWGIbGyaeaadaaeqaqaaiabdgeabnaaBaaaleaacqWGPbqAcqGGSaalcqWGWbaCaeqaaOGaey4kaSIaeGinaqJaemOyaigaleaacqWGPbqAaeqaniabggHiLdaaaOGaeiykaKcaaa@4B15@

where *S *is the scoring matrix, *A *is the frequency matrix, *n *is the nucleotide, *p *is the position within each binding site. The pseudocount, *b*, is set at the relatively small value of 0.25 to allow limited tolerance of base-pairs which have never been observed in a given position for a binding site. When comparing this scoring matrix to a sequence of the same size, adding the scores for the nucleotide that is at that same position in the sequence gives you the log of probability that the sequence matches the model.

The probability that a sequence is not a binding site is based on background dinucleotide frequencies. For each individual species, we went through all promoters and calculated the probability of each dinucleotide transition. For instance, P(AT)=Observed(AT)Observed(Nucleotides)
 MathType@MTEF@5@5@+=feaafiart1ev1aaatCvAUfKttLearuWrP9MDH5MBPbIqV92AaeXatLxBI9gBaebbnrfifHhDYfgasaacH8akY=wiFfYdH8Gipec8Eeeu0xXdbba9frFj0=OqFfea0dXdd9vqai=hGuQ8kuc9pgc9s8qqaq=dirpe0xb9q8qiLsFr0=vr0=vr0dc8meaabaqaciaacaGaaeqabaqabeGadaaakeaacqWGqbaucqGGOaakcqWGbbqqcqWGubavcqGGPaqkcqGH9aqpdaWcaaqaaiabd+eapjabdkgaIjabdohaZjabdwgaLjabdkhaYjabdAha2jabdwgaLjabdsgaKjabcIcaOiabdgeabjabdsfaujabcMcaPaqaaiabd+eapjabdkgaIjabdohaZjabdwgaLjabdkhaYjabdAha2jabdwgaLjabdsgaKjabcIcaOiabd6eaojabdwha1jabdogaJjabdYgaSjabdwgaLjabd+gaVjabdsha0jabdMgaPjabdsgaKjabdwgaLjabdohaZjabcMcaPaaaaaa@5CD2@. We also calculated the probability of observing each nucleotide individually. The probability of observing any sequence can be calculated from those probabilities by multiplying the probability of the first nucleotide by the probability of each nucleotide transition. The log probability can be found by adding the log of each probability.

P(ACT)=P(A)*P(AC)P(A)*P(CT)P(C)log⁡(P(ACT))=log⁡(P(A))+log⁡(P(AC)P(A))+log⁡(P(CT)P(C))
 MathType@MTEF@5@5@+=feaafiart1ev1aaatCvAUfKttLearuWrP9MDH5MBPbIqV92AaeXatLxBI9gBaebbnrfifHhDYfgasaacH8akY=wiFfYdH8Gipec8Eeeu0xXdbba9frFj0=OqFfea0dXdd9vqai=hGuQ8kuc9pgc9s8qqaq=dirpe0xb9q8qiLsFr0=vr0=vr0dc8meaabaqaciaacaGaaeqabaqabeGadaaakqaabeqaaiabdcfaqjabcIcaOiabdgeabjabdoeadjabdsfaujabcMcaPiabg2da9iabdcfaqjabcIcaOiabdgeabjabcMcaPiabcQcaQmaalaaabaGaemiuaaLaeiikaGIaemyqaeKaem4qamKaeiykaKcabaGaemiuaaLaeiikaGIaemyqaeKaeiykaKcaaiabcQcaQmaalaaabaGaemiuaaLaeiikaGIaem4qamKaemivaqLaeiykaKcabaGaemiuaaLaeiikaGIaem4qamKaeiykaKcaaaqaaiGbcYgaSjabc+gaVjabcEgaNjabcIcaOiabdcfaqjabcIcaOiabdgeabjabdoeadjabdsfaujabcMcaPiabcMcaPiabg2da9iGbcYgaSjabc+gaVjabcEgaNjabcIcaOiabdcfaqjabcIcaOiabdgeabjabcMcaPiabcMcaPiabgUcaRiGbcYgaSjabc+gaVjabcEgaNjabcIcaOmaalaaabaGaemiuaaLaeiikaGIaemyqaeKaem4qamKaeiykaKcabaGaemiuaaLaeiikaGIaemyqaeKaeiykaKcaaiabcMcaPiabgUcaRiGbcYgaSjabc+gaVjabcEgaNjabcIcaOmaalaaabaGaemiuaaLaeiikaGIaem4qamKaemivaqLaeiykaKcabaGaemiuaaLaeiikaGIaem4qamKaeiykaKcaaiabcMcaPaaaaa@8193@

The goal of this study was to create a comprehensive list of possible transcription factor targets. The log of the sequence length is often subtracted to correct for the number of possible sites being searched. While subtracting by the full log of the sequence length would provide a more rigorous control, we deliberately chose to increase the sensitivity of the method at the expense of specificity. This increases the number of targets found while decreasing the average quality of the target genes, allowing us to better take advantage of homology to eliminate false positives. Instead of subtracting the log of the sequence length, we subtracted a smaller "correction value". The larger the correction value, the more stringent the search is. To analyze the effects of decreasing specificity on the quality of the target gene set, we use a measure called the positive predicted value. Intuitively, this measure is the probability that a predicted site is a true positive. The positive predictive value is defined as TruePositivesFalsePositives+TruePositives
 MathType@MTEF@5@5@+=feaafiart1ev1aaatCvAUfKttLearuWrP9MDH5MBPbIqV92AaeXatLxBI9gBaebbnrfifHhDYfgasaacH8akY=wiFfYdH8Gipec8Eeeu0xXdbba9frFj0=OqFfea0dXdd9vqai=hGuQ8kuc9pgc9s8qqaq=dirpe0xb9q8qiLsFr0=vr0=vr0dc8meaabaqaciaacaGaaeqabaqabeGadaaakeaadaWcaaqaaiabdsfaujabdkhaYjabdwha1jabdwgaLjabdcfaqjabd+gaVjabdohaZjabdMgaPjabdsha0jabdMgaPjabdAha2jabdwgaLjabdohaZbqaaiabdAeagjabdggaHjabdYgaSjabdohaZjabdwgaLjabdcfaqjabd+gaVjabdohaZjabdMgaPjabdsha0jabdMgaPjabdAha2jabdwgaLjabdohaZjabgUcaRiabdsfaujabdkhaYjabdwha1jabdwgaLjabdcfaqjabd+gaVjabdohaZjabdMgaPjabdsha0jabdMgaPjabdAha2jabdwgaLjabdohaZbaaaaa@6400@.

In terms of our data, the positive predictive value is

ObservedSites−ExpectedSitesObservedSites
 MathType@MTEF@5@5@+=feaafiart1ev1aaatCvAUfKttLearuWrP9MDH5MBPbIqV92AaeXatLxBI9gBaebbnrfifHhDYfgasaacH8akY=wiFfYdH8Gipec8Eeeu0xXdbba9frFj0=OqFfea0dXdd9vqai=hGuQ8kuc9pgc9s8qqaq=dirpe0xb9q8qiLsFr0=vr0=vr0dc8meaabaqaciaacaGaaeqabaqabeGadaaakeaadaWcaaqaaiabd+eapjabdkgaIjabdohaZjabdwgaLjabdkhaYjabdAha2jabdwgaLjabdsgaKjabdofatjabdMgaPjabdsha0jabdwgaLjabdohaZjabgkHiTiabdweafjabdIha4jabdchaWjabdwgaLjabdogaJjabdsha0jabdwgaLjabdsgaKjabdofatjabdMgaPjabdsha0jabdwgaLjabdohaZbqaaiabd+eapjabdkgaIjabdohaZjabdwgaLjabdkhaYjabdAha2jabdwgaLjabdsgaKjabdofatjabdMgaPjabdsha0jabdwgaLjabdohaZbaaaaa@6204@.

The expected number of sites is the number of sites expected to be conserved if there was no association between a binding site existing in mouse and its human homologue. A series of possible correction values are plotted against the positive predicted value (Additional File 10). Because it is the point at which CREB and zif268 positive predictive values plateau, we chose to use a correction value of 300. The final positive predictive value based on comparative genomics is found in Table 1 under "Homologues." A binding site is considered a hit if the final calculated score is above zero. The equation used to determine the final score is given below:

*Score *= log(*P*(*Model*)) - log(*P*(*Background*)) - log(*correction*_*value*)

### Global data analysis

The positive predictive values for the individual species is calculated by comparing the promoter region to the intergenic region (see Table 1). It is still calculated as ObservedSites−ExpectedSitesObservedSites
 MathType@MTEF@5@5@+=feaafiart1ev1aaatCvAUfKttLearuWrP9MDH5MBPbIqV92AaeXatLxBI9gBaebbnrfifHhDYfgasaacH8akY=wiFfYdH8Gipec8Eeeu0xXdbba9frFj0=OqFfea0dXdd9vqai=hGuQ8kuc9pgc9s8qqaq=dirpe0xb9q8qiLsFr0=vr0=vr0dc8meaabaqaciaacaGaaeqabaqabeGadaaakeaadaWcaaqaaiabd+eapjabdkgaIjabdohaZjabdwgaLjabdkhaYjabdAha2jabdwgaLjabdsgaKjabdofatjabdMgaPjabdsha0jabdwgaLjabdohaZjabgkHiTiabdweafjabdIha4jabdchaWjabdwgaLjabdogaJjabdsha0jabdwgaLjabdsgaKjabdofatjabdMgaPjabdsha0jabdwgaLjabdohaZbqaaiabd+eapjabdkgaIjabdohaZjabdwgaLjabdkhaYjabdAha2jabdwgaLjabdsgaKjabdofatjabdMgaPjabdsha0jabdwgaLjabdohaZbaaaaa@6204@, but the observed sites is now the percentage of promoter targets with a binding site and the expected sites is the percentage of intergenic regions with that binding site.

Associations between co-occurring binding sites were analyzed by applying a 2-tailed Fisher's exact test [79], using a web-based calculator [80], to the 2 by 2 contingency table of counts of occurrence of either, both, or neither site.

Analysis of the function of the activity-dependent transcription factor targets was done using GOstat [81], an online tool for finding overrepresented ontologies in a set of genes [54]. The list of targets for each transcription factor binding site and species was searched against the entire list of promoters for overrepresentation of different gene ontology classes.

The VP16-CREB expression data [37] was obtained from the Gene Expression Omnibus (Barrett), record GSE3965, on the NCBI website. The SOFT data files were converted to a single matrix using the GEOquery as part of the Bioconductor [82] package for R. We separated out the expression data into two groups: a control group where CREB is expressed at normal levels (On dox, rev, wt) and an experimental group where VP16-CREB has been active from one to five weeks (1w, 2w, 5w) [37]. Only experiments where the entire hippocampus is dissected are used, not micro-dissected CA1 regions of the hippocampus, where region-specific gene expression could bias the results. The data was analyzed using the GSEA software package for Windows [64]. The change in gene expression levels between the control and the super active CREB were determined by a signal-to-noise metric. We created genesets out of our conserved CREB targets and the conserved CREB targets identified by Conkright et al. [65]. Symbols present on these lists but with no corresponding microarray probe are ignored. The expression levels corresponding to the symbols in the genesets are queried for enrichment in the control versus the experimental microarray datasets using the methods described in Subramanian et al. A list of genes identified as the leading edge subset [64], which are genes that contribute to the enrichment of the CREB targets in the microarray data, are listed as the overlap between our dataset and VP16-CREB dataset [37].

## Abbreviations

ChIP, chromatin immunoprecipitation; IEG, intermediate early gene; LTP, long term potentiation; PCR, polymerase chain reaction; PSSM, position-specific scoring matrix; PWM, position weight matrix

## Authors' contributions

ARP implemented all computational tools developed for this study and performed all computational and statistical analyses. ALB conceived the project and advised ARP on experimental design, data sets, and analysis. RS participated in the design of the study and advised ARP on computational and statistical issues. All authors read and approved the final manuscript.

## Supplementary Material

Additional File 1 Table of all identified CREB and zif268 target genes in mouse. The file includes all mouse genes that were searched indicating the number of identified CREB or zif268 target sites in each gene. Also noted is the score for each site, the mRNA, the gene ID, and a gene description. Click here for file

Additional File 2 Table of all identified CREB and zif268 target genes in human. The file includes all human genes that were searched indicating the number of identified CREB or zif268 target sites in each gene. Also noted is the score for each site, the mRNA, the gene ID, and a gene description. Click here for file

Additional File 3 Table of all identified CREB and zif268 target genes in rat. The file includes all rat genes that were searched indicating the number of identified CREB or zif268 target sites in each gene. Also noted is the score for each site, the mRNA, the gene ID, and a gene description. Click here for file

Additional File 4 Table of all identified CREB and zif268 target genes in the mouse-human homologene dataset. The file includes all mouse-human homologous genes that were searched, indicating the number of identified CREB or zif268 target sites in each gene. Also noted is the gene ID and a gene description. Click here for file

Additional File 5 Position and sequence of CREB and zif268 binding sites in mouse promoter regions. Mouse genes that had one or more CREB and zif268 binding sites are listed in table format, with the precise nucleotide sequence corresponding to the binding site and the relative position of this sequence within the promoter region indicated. Genes are identified by gene ID, symbol, and gene descriptor. Click here for file

Additional File 6 Position and sequence of CREB and zif268 binding sites in human promoter regions. Human genes that had one or more CREB and zif268 binding sites are listed in table format, with the precise nucleotide sequence corresponding to the binding site and the relative position of this sequence within the promoter region indicated. Genes are identified by gene ID, symbol, and gene descriptor. Click here for file

Additional File 7 Position and sequence of CREB and zif268 binding sites in rat promoter regions. Rat genes that had one or more CREB and zif268 binding sites are listed in table format, with the precise nucleotide sequence corresponding to the binding site and the relative position of this sequence within the promoter region indicated. Genes are identified by gene ID, symbol, and gene descriptor. Click here for file

Additional File 8 Gene functions associated with CREB and zif268 targets. A list of the broad gene ontology classifications where CREB or zif268 targets showed an over- or underrepresentation, according to the GOstat ontology classifier. Click here for file

Additional File 9 CREB or zif268 target genes in overrepresented gene ontology categories. The specific genes that were placed in over- or underrepresented gene onotologies using the GOstat resource are listed according to ontology classification. Click here for file

Additional File 10 Comparative genomics as a metric for transcription factor targets quality. Transcription factor binding site searches were done with varying correction scores that correspond to the specificity of the search. The log of the specificity is subtracted from every subsequence scored by the program to correct for sequence length (see Methods). A higher specificity means a smaller number of higher quality binding sites are used. The predicted fraction of true positives or positive predictive value is defined as (true positives)/(true positives + false positives). This measure is estimated as (observed sites - expected sites)/(observed sites). The observed sites are the targets verified by comparative genomics while the expected sites are the number of binding sites one would find by chance if comparing independent human/mouse datasets. Click here for file

## Figures and Tables

**Table 1:** Scores of computational search for activity-dependent transcription factor binding sites

	Mouse			Human			Homologue^a^		
			
Measure	Promoter^b^	Intergenic^c^	Score^d^	Promoter^b^	Intergenic^c^	Score^d^	Complete^e^	Conserved^f^	Score^g^
Total genes	18071	13475		19794	15178		13365	516	
CREB targets	1050	279	0.64	1389	388	0.64	830	356	0.83
zif268 targets	1382	200	0.81	1203	244	0.74	1136	166	0.56
% CREB genes	5.81%	2.07%		7.02%	2.56%		6.21%	2.66%	
% zif268 genes	7.65%	1.48%		6.08%	1.61%		8.50%	1.24%	

^a ^The homologue dataset was contructed using the Homologene resource on the NCBI website ().

^b ^The promoter region is the area from -1,000 to 200 bp relative to transcription start.

^c ^Intergenic regions refer to an area 50,000 bp away from transcription start. Fewer intergenic regions are available than genes because of gaps in sequencing.

^d ^The score is determined by the positive predictive value which is a conservative estimate of the fraction of found transcription factor binding sites that are true hits. It is calculated as (%Observed - %Intergenic)/(%Observed).

^e^The complete homologue numbers refer the the number of human/mouse homologous pairs identified using the Homologene resources.

^f ^The conserved dataset is a subset of the Homologene dataset that consists of only those genes for which homologous pairs contain a conserved binding site.

^g ^The quality is determined by the positive predictive value which is a conservative estimate of the percentage of found transcription factor binding sites that are true hits. It is calculated as (%Conserved Observed - %Predicted Conserved)/(%Conserved Observed).

**Table 2:** Experimental support for binding site activity

*protein*	*gene name*	*site expected*	*site found*	*Dataset*^a^	*verification*
activity-related cytoskeletal protein	arc	zif268	*none*	n/a^b^	[83]
BDNF	bdnf	CREB	*none*	n/a^c^	[84, 85]
beta-polymerase	polb	CREB	CREB	C	[86]
cathepsin L	ctsl	CREB	CREB	C	[87]
c-fos	fos	CREB	CREB	M	[88, 89]
early response gene 3	egr3	zif268	CREB	M	[90]
fibronectin	fn1	CREB	CREB	M	[91]
glycoprotein hormone alpha	cga	CREB	CREB	H^d^	[62]
Ngfi-A binding protein 2	nab2	zif268	zif268	C	[90]
proteasome subunit, alpha type 5	psma5	zif268	zif268	M	[92]
protein phosphatase 2a, catalytic	ppp2ca	CREB	CREB	C	[93]
S100B	s100b	CREB	CREB	C	[94]
somatostatin	sst	CREB	CREB	C	[58]
steroid 11-beta hydroxylase	cyp11b1	CREB	CREB	C	[95]
synapsin I	syn1	zif268	CREB	M	[96, 97]
synapsin II	syn2	zif268	zif268	C	[61]
transporter associated with antigen processing 2	tap2	zif268	zif268	M	[92]
tropomyosin 1, alpha	tpm1	CREB	CREB+zif268	M	[37]
tyrosine hydroxylase	th	CREB	CREB	C	[59, 60]
vasoactive intestinal peptide	vip	CREB	CREB	C	[98]

^a ^Mouse (M), Human (H), or Conserved (C) homologene dataset where a putative binding site was found

^b^A zif268 site in the mouse arc gene was identified in an earlier version of the search, but following an important correction for GC content in the gene promoter this site was no longer recognized

^c ^The CREB site in promoter I that has been well-characterized in rat (Tabuchi et al. 2002) did not qualify as having a binding site by the quality score criterion used in this analysis

^d ^Glycoprotein hormone alpha has a well documented CREB site in human that is absent in rodents based on a single bp mutation

**Table 3:** Overlapping regulatory targets: comparison with other studies

Symbol	Gene Name	Accession #^a^	Conserved site^b^	Type of study^c^	Reference
Clcn3	chloride channel 3	NM007711	CREB	ChIP/SACO	25
Cycs	cytochrome c, somatic	NM007808	CREB	ChIP/SACO	25
Fn1	fibronectin 1	AK147683	CREB	ChIP/SACO	25
FosB	FosB	NM008036	CREB	ChIP/SACO	25
Ldh1	lactate dehydrogenase 1, A chain	BC066858	CREB	ChIP/SACO	25
Mlf2	myeloid leukemia factor 2	NM145385	CREB	ChIP/SACO	25
Nf1	neurofibromatosis 1	NM010897	CREB	ChIP/SACO	25
Ppargc2	peroxisome proliferative activated receptor, gamma, coactivator 1 alpha	NM008904	CREB	ChIP/SACO	25
Sstr2	somatostatin receptor 2	NM009217	CREB	ChIP/SACO	25
St13	suppression of tumorigenicity 13	NM133726	CREB	ChIP/SACO	25
Socs7	suppressor of cytokine signaling 7	NM138657	CREB	ChIP/SACO	25
TpT1	tumor protein, translationally-controlled 1	NM009429	CREB	ChIP/SACO	25
JunD1	Jun proto-oncogene related gene d1	NM010592, 16478	CREB	ChIP/SACO, CREB overexpression	25, 37
Maff	v-maf musculoaponeurotic fibrosarcoma oncogene family, protein F (avian)	NM_010755, 17133	CREB	ChIP/SACO, CREB overexpression	25, 37
Arg2	arginase type II	11847	CREB	CREB overexpression	37
Avpi1	arginine vasopressin-induced 1	69534	CREB	CREB overexpression	37
Arih1	ariadne ubiquitin-conjugating enzyme E2 binding protein homolog 1 (Drosophila)	23806	CREB	CREB overexpression	37
Cd2ap	CD2-associated protein	12488	CREB	CREB overexpression	37
Dhx36	DEAH (Asp-Glu-Ala-His) box polypeptide 36	72162	CREB	CREB overexpression	37
Dusp1	dual specificity phosphatase 1	19252	CREB	CREB overexpression	37
Ell2	elongation factor RNA polymerase II 2	192657	CREB	CREB overexpression	37
AW050020	expressed sequence AW050020	268420	CREB	CREB overexpression	37
Foxd3	forkhead box D3	15221	CREB	CREB overexpression	37
Gem	GTP binding protein (gene overexpressed in skeletal muscle)	14579	CREB	CREB overexpression	37
Mcm2	minichromosome maintenance deficient 2 mitotin (S. cerevisiae)	17216	CREB	CREB overexpression	37
Mlf1	myeloid leukemia factor 1	17349	CREB	CREB overexpression	37
Myh10	myosin, heavy polypeptide 10, non-muscle	77579	CREB	CREB overexpression	37
Nek1	NIMA (never in mitosis gene a)-related expressed kinase 1	18004	CREB	CREB overexpression	37
Pde7a	phosphodiesterase 7A	18583	CREB	CREB overexpression	37
Psmb8	proteosome (prosome, macropain) subunit, beta type 8 (large multifunctional peptidase 7)	16913	CREB	CREB overexpression	37
2700085E05Rik	RIKEN cDNA 2700085E05 gene	67201	CREB	CREB overexpression	37
4933407C03Rik	RIKEN cDNA 4933407C03 gene	74440	CREB	CREB overexpression	37
6330407G11Rik	RIKEN cDNA 6330407G11 gene	68050	CREB	CREB overexpression	37
Rbms2	RNA binding motif, single stranded interacting protein 2	56516	CREB	CREB overexpression	37
St7	Suppression of tumorigenicity 7	64213	CREB	CREB overexpression	37
Tgif2	TGFB-induced factor 2	228839	CREB	CREB overexpression	37
Trip6	thyroid hormone receptor interactor 6	22051	CREB	CREB overexpression	37
Tle3	transducin-like enhancer of split 3, homolog of Drosophila E(spl)	21887	CREB	CREB overexpression	37
Tpm4	tropomyosin 4	326618	CREB	CREB overexpression	37
Trp53inp2	tumor protein p53 inducible nuclear protein 2	68728	CREB	CREB overexpression	37
Th	tyrosine hydroxylase	21823	CREB	CREB overexpression	37
Wbp5	WW domain binding protein 5	22381	CREB	CREB overexpression	37
Xpr1	xenotropic and polytropic retrovirus receptor 1	19775	CREB	CREB overexpression	37
Znrd1	zinc ribbon domain containing, 1	66136	CREB	CREB overexpression	37
Sgta	small glutamine-rich tetratricopeptide repeat (TPR)-containing, alpha	NM024499	Zif268	Zif268 overexpression	57

^a^Accession numbers for CREB overexpression experiments are the GeneID, not the mRNA acession number.

^b^Gene target had a conserved mouse-human transcription factor binding site in the promoter region and was also identified in the referenced study.

^c^Type of experimental analysis carried out in the referenced study. ChIP indicated chromatin immunoprecipitation followed by a serial analysis of chromatin occupancy in DNA isolated from rat PC12 cells that had been stimulated with forskolin to increase intracellular cAMP 15 minutes prior to DNA extraction [25]. Zif268 overexpression refers to studies where zif268 was overexpressed in PC12 cells, and RNA was extracted 48 hrs post-transfection [57]. CREB overexpression refers to studies where a super-active form of CREB is expressed in mice for 1 to 5 weeks. RNA is extracted from hippocampus and run on Affymetrix GeneChip Murine Genome U74 Version 2 Set MG-U74A [37]. Our analysis of the data was done using GSEA as described in the methods.

## References

[B1] Deisseroth K, Mermelstein PG, Xia H, Tsien RW (2003). Signaling from synapse to nucleus: the logic behind the mechanisms. Curr Opin Neurobiol.

[B2] Shaywitz AJ, Greenberg ME (1999). CREB: a stimulus-induced transcription factor activated by a diverse array of extracellular signals. Annu Rev Biochem.

[B3] Zhao M, Adams JP, Dudek SM (2005). Pattern-dependent role of NMDA receptors in action potential generation: consequences on extracellular signal-regulated kinase activation. J Neurosci.

[B4] Dash PK, Hochner B, Kandel ER (1990). Injection of the cAMP-responsive element into the nucleus of Aplysia sensory neurons blocks long-term facilitation. Nature.

[B5] Jones MW, Errington ML, French PJ, Fine A, Bliss TV, Garel S, Charnay P, Bozon B, Laroche S, Davis S (2001). A requirement for the immediate early gene Zif268 in the expression of late LTP and long-term memories. Nat Neurosci.

[B6] Elliott RC, Lowenstein DH (2004). Gene expression profiling of seizure disorders. Neurochem Res.

[B7] Rakhade SN, Yao B, Ahmed S, Asano E, Beaumont TL, Shah AK, Draghici S, Krauss R, Chugani HT, Sood S, Loeb JA (2005). A common pattern of persistent gene activation in human neocortical epileptic foci. Ann Neurol.

[B8] Hong EJ, West AE, Greenberg ME (2005). Transcriptional control of cognitive development. Curr Opin Neurobiol.

[B9] Befort K, Karchewski L, Lanoue C, Woolf CJ (2003). Selective up-regulation of the growth arrest DNA damage-inducible gene Gadd45 alpha in sensory and motor neurons after peripheral nerve injury. Eur J Neurosci.

[B10] Costigan M, Befort K, Karchewski L, Griffin RS, D'Urso D, Allchorne A, Sitarski J, Mannion JW, Pratt RE, Woolf CJ (2002). Replicate high-density rat genome oligonucleotide microarrays reveal hundreds of regulated genes in the dorsal root ganglion after peripheral nerve injury. BMC Neurosci.

[B11] Laifenfeld D, Klein E, Ben-Shachar D (2002). Norepinephrine alters the expression of genes involved in neuronal sprouting and differentiation: relevance for major depression and antidepressant mechanisms. J Neurochem.

[B12] Lee KH, Ryu CJ, Hong HJ, Kim J, Lee EH (2005). CDNA microarray analysis of nerve growth factor-regulated gene expression profile in rat PC12 cells. Neurochem Res.

[B13] Luo Y, Long JM, Spangler EL, Longo DL, Ingram DK, Weng NP (2001). Identification of maze learning-associated genes in rat hippocampus by cDNA microarray. J Mol Neurosci.

[B14] Valerio A, Ferrario M, Martinez FO, Locati M, Ghisi V, Bresciani LG, Mantovani A, Spano P (2004). Gene expression profile activated by the chemokine CCL5/RANTES in human neuronal cells. J Neurosci Res.

[B15] Yao WD, Gainetdinov RR, Arbuckle MI, Sotnikova TD, Cyr M, Beaulieu JM, Torres GE, Grant SG, Caron MG (2004). Identification of PSD-95 as a regulator of dopamine-mediated synaptic and behavioral plasticity. Neuron.

[B16] Del Rio JA, Barlow C (2002). Genomics and neurological phenotypes: applications for seizure-induced damage. Prog Brain Res.

[B17] Flood WD, Moyer RW, Tsykin A, Sutherland GR, Koblar SA (2004). Nxf and Fbxo33: novel seizure-responsive genes in mice. Eur J Neurosci.

[B18] Hunsberger JG, Bennett AH, Selvanayagam E, Duman RS, Newton SS (2005). Gene profiling the response to kainic acid induced seizures. Brain Res Mol Brain Res.

[B19] Lukasiuk K, Pitkanen A (2004). Large-scale analysis of gene expression in epilepsy research: is synthesis already possible?. Neurochem Res.

[B20] Tang Y, Lu A, Aronow BJ, Sharp FR (2001). Blood genomic responses differ after stroke, seizures, hypoglycemia, and hypoxia: blood genomic fingerprints of disease. Ann Neurol.

[B21] Wilson DN, Chung H, Elliott RC, Bremer E, George D, Koh S (2005). Microarray analysis of postictal transcriptional regulation of neuropeptides. J Mol Neurosci.

[B22] Majdan MS (2006). Effects of visual experience on activity-dependent gene regulation in cortex.. Nature Neuroscience.

[B23] Tropea D, Kreiman G, Lyckman A, Mukherjee S, Yu H, Horng S, Sur M (2006). Gene expression changes and molecular pathways mediating activity-dependent plasticity in visual cortex. Nat Neurosci.

[B24] Hakimi MA, Bochar DA, Chenoweth J, Lane WS, Mandel G, Shiekhattar R (2002). A core-BRAF35 complex containing histone deacetylase mediates repression of neuronal-specific genes. Proc Natl Acad Sci U S A.

[B25] Impey S, McCorkle SR, Cha-Molstad H, Dwyer JM, Yochum GS, Boss JM, McWeeney S, Dunn JJ, Mandel G, Goodman RH (2004). Defining the CREB regulon: a genome-wide analysis of transcription factor regulatory regions. Cell.

[B26] Israsena N, Hu M, Fu W, Kan L, Kessler JA (2004). The presence of FGF2 signaling determines whether beta-catenin exerts effects on proliferation or neuronal differentiation of neural stem cells. Dev Biol.

[B27] Sun YM, Greenway DJ, Johnson R, Street M, Belyaev ND, Deuchars J, Bee T, Wilde S, Buckley NJ (2005). Distinct Profiles of REST Interactions with Its Target Genes at Different Stages of Neuronal Development. Mol Biol Cell.

[B28] Vanderluit JL, Ferguson KL, Nikoletopoulou V, Parker M, Ruzhynsky V, Alexson T, McNamara SM, Park DS, Rudnicki M, Slack RS (2004). p107 regulates neural precursor cells in the mammalian brain. J Cell Biol.

[B29] Cha-Molstad H, Keller DM, Yochum GS, Impey S, Goodman RH (2004). Cell-type-specific binding of the transcription factor CREB to the cAMP-response element. Proc Natl Acad Sci U S A.

[B30] Amadio M, Govoni S, Alkon DL, Pascale A (2004). Emerging targets for the pharmacology of learning and memory. Pharmacol Res.

[B31] Hofmann HA (2003). Functional genomics of neural and behavioral plasticity. J Neurobiol.

[B32] Bulyk ML (2003). Computational prediction of transcription-factor binding site locations. Genome Biol.

[B33] Qiu P (2003). Recent advances in computational promoter analysis in understanding the transcriptional regulatory network. Biochem Biophys Res Commun.

[B34] Vavouri T, Elgar G (2005). Prediction of cis-regulatory elements using binding site matrices--the successes, the failures and the reasons for both. Curr Opin Genet Dev.

[B35] Herdegen T, Leah JD (1998). Inducible and constitutive transcription factors in the mammalian nervous system: control of gene expression by Jun, Fos and Krox, and CREB/ATF proteins. Brain Res Brain Res Rev.

[B36] Yin JC, Wallach JS, Del Vecchio M, Wilder EL, Zhou H, Quinn WG, Tully T (1994). Induction of a dominant negative CREB transgene specifically blocks long-term memory in Drosophila. Cell.

[B37] Barco A, Patterson S, Alarcon JM, Gromova P, Mata-Roig M, Morozov A, Kandel ER (2005). Gene expression profiling of facilitated L-LTP in VP16-CREB mice reveals that BDNF is critical for the maintenance of LTP and its synaptic capture. Neuron.

[B38] Bourtchuladze R, Frenguelli B, Blendy J, Cioffi D, Schutz G, Silva AJ (1994). Deficient long-term memory in mice with a targeted mutation of the cAMP-responsive element-binding protein. Cell.

[B39] Beckmann AM, Wilce PA (1997). Egr transcription factors in the nervous system. Neurochem Int.

[B40] Valjent E, Aubier B, Corbille AG, Brami-Cherrier K, Caboche J, Topilko P, Girault JA, Herve D (2006). Plasticity-associated gene Krox24/Zif268 is required for long-lasting behavioral effects of cocaine. J Neurosci.

[B41] Corriveau RA, Huh GS, Shatz CJ (1998). Regulation of class I MHC gene expression in the developing and mature CNS by neural activity. Neuron.

[B42] Guan Z, Saraswati S, Adolfsen B, Littleton JT (2005). Genome-wide transcriptional changes associated with enhanced activity in the Drosophila nervous system. Neuron.

[B43] Nedivi E, Hevroni D, Naot D, Israeli D, Citri Y (1993). Numerous candidate plasticity-related genes revealed by differential cDNA cloning. Nature.

[B44] Benbrook DM, Jones NC (1994). Different binding specificities and transactivation of variant CRE's by CREB complexes. Nucleic Acids Res.

[B45] Swirnoff AH, Milbrandt J (1995). DNA-binding specificity of NGFI-A and related zinc finger transcription factors. Mol Cell Biol.

[B46] Zhang X, Odom DT, Koo SH, Conkright MD, Canettieri G, Best J, Chen H, Jenner R, Herbolsheimer E, Jacobsen E, Kadam S, Ecker JR, Emerson B, Hogenesch JB, Unterman T, Young RA, Montminy M (2005). Genome-wide analysis of cAMP-response element binding protein occupancy, phosphorylation, and target gene activation in human tissues. Proc Natl Acad Sci U S A.

[B47] Vo N, Klein ME, Varlamova O, Keller DM, Yamamoto T, Goodman RH, Impey S (2005). A cAMP-response element binding protein-induced microRNA regulates neuronal morphogenesis. Proc Natl Acad Sci U S A.

[B48] Wingender E, Chen X, Hehl R, Karas H, Liebich I, Matys V, Meinhardt T, Pruss M, Reuter I, Schacherer F (2000). TRANSFAC: an integrated system for gene expression regulation. Nucleic Acids Res.

[B49] Lander ES, Linton LM, Birren B, Nusbaum C, Zody MC, Baldwin J, Devon K, Dewar K, Doyle M, FitzHugh W, Funke R, Gage D, Harris K, Heaford A, Howland J, Kann L, Lehoczky J, LeVine R, McEwan P, McKernan K, Meldrim J, Mesirov JP, Miranda C, Morris W, Naylor J, Raymond C, Rosetti M, Santos R, Sheridan A, Sougnez C, Stange-Thomann N, Stojanovic N, Subramanian A, Wyman D, Rogers J, Sulston J, Ainscough R, Beck S, Bentley D, Burton J, Clee C, Carter N, Coulson A, Deadman R, Deloukas P, Dunham A, Dunham I, Durbin R, French L, Grafham D, Gregory S, Hubbard T, Humphray S, Hunt A, Jones M, Lloyd C, McMurray A, Matthews L, Mercer S, Milne S, Mullikin JC, Mungall A, Plumb R, Ross M, Shownkeen R, Sims S, Waterston RH, Wilson RK, Hillier LW, McPherson JD, Marra MA, Mardis ER, Fulton LA, Chinwalla AT, Pepin KH, Gish WR, Chissoe SL, Wendl MC, Delehaunty KD, Miner TL, Delehaunty A, Kramer JB, Cook LL, Fulton RS, Johnson DL, Minx PJ, Clifton SW, Hawkins T, Branscomb E, Predki P, Richardson P, Wenning S, Slezak T, Doggett N, Cheng JF, Olsen A, Lucas S, Elkin C, Uberbacher E, Frazier M, Gibbs RA, Muzny DM, Scherer SE, Bouck JB, Sodergren EJ, Worley KC, Rives CM, Gorrell JH, Metzker ML, Naylor SL, Kucherlapati RS, Nelson DL, Weinstock GM, Sakaki Y, Fujiyama A, Hattori M, Yada T, Toyoda A, Itoh T, Kawagoe C, Watanabe H, Totoki Y, Taylor T, Weissenbach J, Heilig R, Saurin W, Artiguenave F, Brottier P, Bruls T, Pelletier E, Robert C, Wincker P, Smith DR, Doucette-Stamm L, Rubenfield M, Weinstock K, Lee HM, Dubois J, Rosenthal A, Platzer M, Nyakatura G, Taudien S, Rump A, Yang H, Yu J, Wang J, Huang G, Gu J, Hood L, Rowen L, Madan A, Qin S, Davis RW, Federspiel NA, Abola AP, Proctor MJ, Myers RM, Schmutz J, Dickson M, Grimwood J, Cox DR, Olson MV, Kaul R, Raymond C, Shimizu N, Kawasaki K, Minoshima S, Evans GA, Athanasiou M, Schultz R, Roe BA, Chen F, Pan H, Ramser J, Lehrach H, Reinhardt R, McCombie WR, de la Bastide M, Dedhia N, Blocker H, Hornischer K, Nordsiek G, Agarwala R, Aravind L, Bailey JA, Bateman A, Batzoglou S, Birney E, Bork P, Brown DG, Burge CB, Cerutti L, Chen HC, Church D, Clamp M, Copley RR, Doerks T, Eddy SR, Eichler EE, Furey TS, Galagan J, Gilbert JG, Harmon C, Hayashizaki Y, Haussler D, Hermjakob H, Hokamp K, Jang W, Johnson LS, Jones TA, Kasif S, Kaspryzk A, Kennedy S, Kent WJ, Kitts P, Koonin EV, Korf I, Kulp D, Lancet D, Lowe TM, McLysaght A, Mikkelsen T, Moran JV, Mulder N, Pollara VJ, Ponting CP, Schuler G, Schultz J, Slater G, Smit AF, Stupka E, Szustakowski J, Thierry-Mieg D, Thierry-Mieg J, Wagner L, Wallis J, Wheeler R, Williams A, Wolf YI, Wolfe KH, Yang SP, Yeh RF, Collins F, Guyer MS, Peterson J, Felsenfeld A, Wetterstrand KA, Patrinos A, Morgan MJ, de Jong P, Catanese JJ, Osoegawa K, Shizuya H, Choi S, Chen YJ (2001). Initial sequencing and analysis of the human genome. Nature.

[B50] Ota T, Suzuki Y, Nishikawa T, Otsuki T, Sugiyama T, Irie R, Wakamatsu A, Hayashi K, Sato H, Nagai K, Kimura K, Makita H, Sekine M, Obayashi M, Nishi T, Shibahara T, Tanaka T, Ishii S, Yamamoto J, Saito K, Kawai Y, Isono Y, Nakamura Y, Nagahari K, Murakami K, Yasuda T, Iwayanagi T, Wagatsuma M, Shiratori A, Sudo H, Hosoiri T, Kaku Y, Kodaira H, Kondo H, Sugawara M, Takahashi M, Kanda K, Yokoi T, Furuya T, Kikkawa E, Omura Y, Abe K, Kamihara K, Katsuta N, Sato K, Tanikawa M, Yamazaki M, Ninomiya K, Ishibashi T, Yamashita H, Murakawa K, Fujimori K, Tanai H, Kimata M, Watanabe M, Hiraoka S, Chiba Y, Ishida S, Ono Y, Takiguchi S, Watanabe S, Yosida M, Hotuta T, Kusano J, Kanehori K, Takahashi-Fujii A, Hara H, Tanase TO, Nomura Y, Togiya S, Komai F, Hara R, Takeuchi K, Arita M, Imose N, Musashino K, Yuuki H, Oshima A, Sasaki N, Aotsuka S, Yoshikawa Y, Matsunawa H, Ichihara T, Shiohata N, Sano S, Moriya S, Momiyama H, Satoh N, Takami S, Terashima Y, Suzuki O, Nakagawa S, Senoh A, Mizoguchi H, Goto Y, Shimizu F, Wakebe H, Hishigaki H, Watanabe T, Sugiyama A, Takemoto M, Kawakami B, Yamazaki M, Watanabe K, Kumagai A, Itakura S, Fukuzumi Y, Fujimori Y, Komiyama M, Tashiro H, Tanigami A, Fujiwara T, Ono T, Yamada K, Fujii Y, Ozaki K, Hirao M, Ohmori Y, Kawabata A, Hikiji T, Kobatake N, Inagaki H, Ikema Y, Okamoto S, Okitani R, Kawakami T, Noguchi S, Itoh T, Shigeta K, Senba T, Matsumura K, Nakajima Y, Mizuno T, Morinaga M, Sasaki M, Togashi T, Oyama M, Hata H, Watanabe M, Komatsu T, Mizushima-Sugano J, Satoh T, Shirai Y, Takahashi Y, Nakagawa K, Okumura K, Nagase T, Nomura N, Kikuchi H, Masuho Y, Yamashita R, Nakai K, Yada T, Nakamura Y, Ohara O, Isogai T, Sugano S (2004). Complete sequencing and characterization of 21,243 full-length human cDNAs. Nat Genet.

[B51] Venter JC, Adams MD, Myers EW, Li PW, Mural RJ, Sutton GG, Smith HO, Yandell M, Evans CA, Holt RA, Gocayne JD, Amanatides P, Ballew RM, Huson DH, Wortman JR, Zhang Q, Kodira CD, Zheng XH, Chen L, Skupski M, Subramanian G, Thomas PD, Zhang J, Gabor Miklos GL, Nelson C, Broder S, Clark AG, Nadeau J, McKusick VA, Zinder N, Levine AJ, Roberts RJ, Simon M, Slayman C, Hunkapiller M, Bolanos R, Delcher A, Dew I, Fasulo D, Flanigan M, Florea L, Halpern A, Hannenhalli S, Kravitz S, Levy S, Mobarry C, Reinert K, Remington K, Abu-Threideh J, Beasley E, Biddick K, Bonazzi V, Brandon R, Cargill M, Chandramouliswaran I, Charlab R, Chaturvedi K, Deng Z, Di Francesco V, Dunn P, Eilbeck K, Evangelista C, Gabrielian AE, Gan W, Ge W, Gong F, Gu Z, Guan P, Heiman TJ, Higgins ME, Ji RR, Ke Z, Ketchum KA, Lai Z, Lei Y, Li Z, Li J, Liang Y, Lin X, Lu F, Merkulov GV, Milshina N, Moore HM, Naik AK, Narayan VA, Neelam B, Nusskern D, Rusch DB, Salzberg S, Shao W, Shue B, Sun J, Wang Z, Wang A, Wang X, Wang J, Wei M, Wides R, Xiao C, Yan C, Yao A, Ye J, Zhan M, Zhang W, Zhang H, Zhao Q, Zheng L, Zhong F, Zhong W, Zhu S, Zhao S, Gilbert D, Baumhueter S, Spier G, Carter C, Cravchik A, Woodage T, Ali F, An H, Awe A, Baldwin D, Baden H, Barnstead M, Barrow I, Beeson K, Busam D, Carver A, Center A, Cheng ML, Curry L, Danaher S, Davenport L, Desilets R, Dietz S, Dodson K, Doup L, Ferriera S, Garg N, Gluecksmann A, Hart B, Haynes J, Haynes C, Heiner C, Hladun S, Hostin D, Houck J, Howland T, Ibegwam C, Johnson J, Kalush F, Kline L, Koduru S, Love A, Mann F, May D, McCawley S, McIntosh T, McMullen I, Moy M, Moy L, Murphy B, Nelson K, Pfannkoch C, Pratts E, Puri V, Qureshi H, Reardon M, Rodriguez R, Rogers YH, Romblad D, Ruhfel B, Scott R, Sitter C, Smallwood M, Stewart E, Strong R, Suh E, Thomas R, Tint NN, Tse S, Vech C, Wang G, Wetter J, Williams S, Williams M, Windsor S, Winn-Deen E, Wolfe K, Zaveri J, Zaveri K, Abril JF, Guigo R, Campbell MJ, Sjolander KV, Karlak B, Kejariwal A, Mi H, Lazareva B, Hatton T, Narechania A, Diemer K, Muruganujan A, Guo N, Sato S, Bafna V, Istrail S, Lippert R, Schwartz R, Walenz B, Yooseph S, Allen D, Basu A, Baxendale J, Blick L, Caminha M, Carnes-Stine J, Caulk P, Chiang YH, Coyne M, Dahlke C, Mays A, Dombroski M, Donnelly M, Ely D, Esparham S, Fosler C, Gire H, Glanowski S, Glasser K, Glodek A, Gorokhov M, Graham K, Gropman B, Harris M, Heil J, Henderson S, Hoover J, Jennings D, Jordan C, Jordan J, Kasha J, Kagan L, Kraft C, Levitsky A, Lewis M, Liu X, Lopez J, Ma D, Majoros W, McDaniel J, Murphy S, Newman M, Nguyen T, Nguyen N, Nodell M, Pan S, Peck J, Peterson M, Rowe W, Sanders R, Scott J, Simpson M, Smith T, Sprague A, Stockwell T, Turner R, Venter E, Wang M, Wen M, Wu D, Wu M, Xia A, Zandieh A, Zhu X (2001). The sequence of the human genome. Science.

[B52] Wheeler DL, Barrett T, Benson DA, Bryant SH, Canese K, Church DM, DiCuccio M, Edgar R, Federhen S, Helmberg W, Kenton DL, Khovayko O, Lipman DJ, Madden TL, Maglott DR, Ostell J, Pontius JU, Pruitt KD, Schuler GD, Schriml LM, Sequeira E, Sherry ST, Sirotkin K, Starchenko G, Suzek TO, Tatusov R, Tatusova TA, Wagner L, Yaschenko E (2005). Database resources of the National Center for Biotechnology Information. Nucleic Acids Res.

[B53] Xu W, Angelis K, Danielpour D, Haddad MM, Bischof O, Campisi J, Stavnezer E, Medrano EE (2000). Ski acts as a co-repressor with Smad2 and Smad3 to regulate the response to type beta transforming growth factor. Proc Natl Acad Sci U S A.

[B54] Beissbarth T, Speed TP (2004). GOstat: find statistically overrepresented Gene Ontologies within a group of genes. Bioinformatics.

[B55] Heurteaux C, Guy N, Laigle C, Blondeau N, Duprat F, Mazzuca M, Lang-Lazdunski L, Widmann C, Zanzouri M, Romey G, Lazdunski M (2004). TREK-1, a K+ channel involved in neuroprotection and general anesthesia. Embo J.

[B56] James AB, Conway AM, Morris BJ (2005). Genomic profiling of the neuronal target genes of the plasticity-related transcription factor -- Zif268. J Neurochem.

[B57] Mayr B, Montminy M (2001). Transcriptional regulation by the phosphorylation-dependent factor CREB. Nat Rev Mol Cell Biol.

[B58] Montminy MR, Bilezikjian LM (1987). Binding of a nuclear protein to the cyclic-AMP response element of the somatostatin gene. Nature.

[B59] Iwata N, Kobayashi K, Sasaoka T, Hidaka H, Nagatsu T (1992). Structure of the mouse tyrosine hydroxylase gene. Biochem Biophys Res Commun.

[B60] Kim KS, Lee MK, Carroll J, Joh TH (1993). Both the basal and inducible transcription of the tyrosine hydroxylase gene are dependent upon a cAMP response element. J Biol Chem.

[B61] Petersohn D, Schoch S, Brinkmann DR, Thiel G (1995). The human synapsin II gene promoter. Possible role for the transcription factor zif268/egr-1, polyoma enhancer activator 3, and AP2. J Biol Chem.

[B62] Nilson JH, Bokar JA, Clay CM, Farmerie TA, Fenstermaker RA, Hamernik DL, Keri RA (1991). Different combinations of regulatory elements may explain why placenta-specific expression of the glycoprotein hormone alpha-subunit gene occurs only in primates and horses. Biol Reprod.

[B63] Subramanian A, Tamayo P, Mootha VK, Mukherjee S, Ebert BL, Gillette MA, Paulovich A, Pomeroy SL, Golub TR, Lander ES, Mesirov JP (2005). Gene set enrichment analysis: a knowledge-based approach for interpreting genome-wide expression profiles. Proc Natl Acad Sci U S A.

[B64] Conkright MD, Guzman E, Flechner L, Su AI, Hogenesch JB, Montminy M (2003). Genome-wide analysis of CREB target genes reveals a core promoter requirement for cAMP responsiveness. Mol Cell.

[B65] Fu M, Zhu X, Zhang J, Liang J, Lin Y, Zhao L, Ehrengruber MU, Chen YE (2003). Egr-1 target genes in human endothelial cells identified by microarray analysis. Gene.

[B66] Svaren J, Ehrig T, Abdulkadir SA, Ehrengruber MU, Watson MA, Milbrandt J (2000). EGR1 target genes in prostate carcinoma cells identified by microarray analysis. J Biol Chem.

[B67] Virolle T, Krones-Herzig A, Baron V, De Gregorio G, Adamson ED, Mercola D (2003). Egr1 promotes growth and survival of prostate cancer cells. Identification of novel Egr1 target genes. J Biol Chem.

[B68] Elemento O, Tavazoie S (2005). Fast and systematic genome-wide discovery of conserved regulatory elements using a non-alignment based approach. Genome Biol.

[B69] Bulyk ML, McGuire AM, Masuda N, Church GM (2004). A motif co-occurrence approach for genome-wide prediction of transcription-factor-binding sites in Escherichia coli. Genome Res.

[B70] Luscombe NM, Babu MM, Yu H, Snyder M, Teichmann SA, Gerstein M (2004). Genomic analysis of regulatory network dynamics reveals large topological changes. Nature.

[B71] Waterston RH, Lindblad-Toh K, Birney E, Rogers J, Abril JF, Agarwal P, Agarwala R, Ainscough R, Alexandersson M, An P, Antonarakis SE, Attwood J, Baertsch R, Bailey J, Barlow K, Beck S, Berry E, Birren B, Bloom T, Bork P, Botcherby M, Bray N, Brent MR, Brown DG, Brown SD, Bult C, Burton J, Butler J, Campbell RD, Carninci P, Cawley S, Chiaromonte F, Chinwalla AT, Church DM, Clamp M, Clee C, Collins FS, Cook LL, Copley RR, Coulson A, Couronne O, Cuff J, Curwen V, Cutts T, Daly M, David R, Davies J, Delehaunty KD, Deri J, Dermitzakis ET, Dewey C, Dickens NJ, Diekhans M, Dodge S, Dubchak I, Dunn DM, Eddy SR, Elnitski L, Emes RD, Eswara P, Eyras E, Felsenfeld A, Fewell GA, Flicek P, Foley K, Frankel WN, Fulton LA, Fulton RS, Furey TS, Gage D, Gibbs RA, Glusman G, Gnerre S, Goldman N, Goodstadt L, Grafham D, Graves TA, Green ED, Gregory S, Guigo R, Guyer M, Hardison RC, Haussler D, Hayashizaki Y, Hillier LW, Hinrichs A, Hlavina W, Holzer T, Hsu F, Hua A, Hubbard T, Hunt A, Jackson I, Jaffe DB, Johnson LS, Jones M, Jones TA, Joy A, Kamal M, Karlsson EK, Karolchik D, Kasprzyk A, Kawai J, Keibler E, Kells C, Kent WJ, Kirby A, Kolbe DL, Korf I, Kucherlapati RS, Kulbokas EJ, Kulp D, Landers T, Leger JP, Leonard S, Letunic I, Levine R, Li J, Li M, Lloyd C, Lucas S, Ma B, Maglott DR, Mardis ER, Matthews L, Mauceli E, Mayer JH, McCarthy M, McCombie WR, McLaren S, McLay K, McPherson JD, Meldrim J, Meredith B, Mesirov JP, Miller W, Miner TL, Mongin E, Montgomery KT, Morgan M, Mott R, Mullikin JC, Muzny DM, Nash WE, Nelson JO, Nhan MN, Nicol R, Ning Z, Nusbaum C, O'Connor MJ, Okazaki Y, Oliver K, Overton-Larty E, Pachter L, Parra G, Pepin KH, Peterson J, Pevzner P, Plumb R, Pohl CS, Poliakov A, Ponce TC, Ponting CP, Potter S, Quail M, Reymond A, Roe BA, Roskin KM, Rubin EM, Rust AG, Santos R, Sapojnikov V, Schultz B, Schultz J, Schwartz MS, Schwartz S, Scott C, Seaman S, Searle S, Sharpe T, Sheridan A, Shownkeen R, Sims S, Singer JB, Slater G, Smit A, Smith DR, Spencer B, Stabenau A, Stange-Thomann N, Sugnet C, Suyama M, Tesler G, Thompson J, Torrents D, Trevaskis E, Tromp J, Ucla C, Ureta-Vidal A, Vinson JP, Von Niederhausern AC, Wade CM, Wall M, Weber RJ, Weiss RB, Wendl MC, West AP, Wetterstrand K, Wheeler R, Whelan S, Wierzbowski J, Willey D, Williams S, Wilson RK, Winter E, Worley KC, Wyman D, Yang S, Yang SP, Zdobnov EM, Zody MC, Lander ES (2002). Initial sequencing and comparative analysis of the mouse genome. Nature.

[B72] Gibbs RA, Weinstock GM, Metzker ML, Muzny DM, Sodergren EJ, Scherer S, Scott G, Steffen D, Worley KC, Burch PE, Okwuonu G, Hines S, Lewis L, DeRamo C, Delgado O, Dugan-Rocha S, Miner G, Morgan M, Hawes A, Gill R, Holt RA, Adams MD, Amanatides PG, Baden-Tillson H, Barnstead M, Chin S, Evans CA, Ferriera S, Fosler C, Glodek A, Gu Z, Jennings D, Kraft CL, Nguyen T, Pfannkoch CM, Sitter C, Sutton GG, Venter JC, Woodage T, Smith D, Lee HM, Gustafson E, Cahill P, Kana A, Doucette-Stamm L, Weinstock K, Fechtel K, Weiss RB, Dunn DM, Green ED, Blakesley RW, Bouffard GG, De Jong PJ, Osoegawa K, Zhu B, Marra M, Schein J, Bosdet I, Fjell C, Jones S, Krzywinski M, Mathewson C, Siddiqui A, Wye N, McPherson J, Zhao S, Fraser CM, Shetty J, Shatsman S, Geer K, Chen Y, Abramzon S, Nierman WC, Havlak PH, Chen R, Durbin KJ, Egan A, Ren Y, Song XZ, Li B, Liu Y, Qin X, Cawley S, Worley KC, Cooney AJ, D'Souza LM, Martin K, Wu JQ, Gonzalez-Garay ML, Jackson AR, Kalafus KJ, McLeod MP, Milosavljevic A, Virk D, Volkov A, Wheeler DA, Zhang Z, Bailey JA, Eichler EE, Tuzun E, Birney E, Mongin E, Ureta-Vidal A, Woodwark C, Zdobnov E, Bork P, Suyama M, Torrents D, Alexandersson M, Trask BJ, Young JM, Huang H, Wang H, Xing H, Daniels S, Gietzen D, Schmidt J, Stevens K, Vitt U, Wingrove J, Camara F, Mar Alba M, Abril JF, Guigo R, Smit A, Dubchak I, Rubin EM, Couronne O, Poliakov A, Hubner N, Ganten D, Goesele C, Hummel O, Kreitler T, Lee YA, Monti J, Schulz H, Zimdahl H, Himmelbauer H, Lehrach H, Jacob HJ, Bromberg S, Gullings-Handley J, Jensen-Seaman MI, Kwitek AE, Lazar J, Pasko D, Tonellato PJ, Twigger S, Ponting CP, Duarte JM, Rice S, Goodstadt L, Beatson SA, Emes RD, Winter EE, Webber C, Brandt P, Nyakatura G, Adetobi M, Chiaromonte F, Elnitski L, Eswara P, Hardison RC, Hou M, Kolbe D, Makova K, Miller W, Nekrutenko A, Riemer C, Schwartz S, Taylor J, Yang S, Zhang Y, Lindpaintner K, Andrews TD, Caccamo M, Clamp M, Clarke L, Curwen V, Durbin R, Eyras E, Searle SM, Cooper GM, Batzoglou S, Brudno M, Sidow A, Stone EA, Venter JC, Payseur BA, Bourque G, Lopez-Otin C, Puente XS, Chakrabarti K, Chatterji S, Dewey C, Pachter L, Bray N, Yap VB, Caspi A, Tesler G, Pevzner PA, Haussler D, Roskin KM, Baertsch R, Clawson H, Furey TS, Hinrichs AS, Karolchik D, Kent WJ, Rosenbloom KR, Trumbower H, Weirauch M, Cooper DN, Stenson PD, Ma B, Brent M, Arumugam M, Shteynberg D, Copley RR, Taylor MS, Riethman H, Mudunuri U, Peterson J, Guyer M, Felsenfeld A, Old S, Mockrin S, Collins F, Celera (2004). Genome sequence of the Brown Norway rat yields insights into mammalian evolution. Nature.

[B73] (http://hgdownload.cse.ucsc.edu/downloads.html)

[B74] GuhaThakurta D, Stormo GD (2001). Identifying target sites for cooperatively binding factors. Bioinformatics.

[B75] Stormo GD (1990). Consensus patterns in DNA. Methods Enzymol.

[B76] Suzuki Y, Yamashita R, Shirota M, Sakakibara Y, Chiba J, Mizushima-Sugano J, Kel AE, Arakawa T, Carninci P, Kawai J, Hayashizaki Y, Takagi T, Nakai K, Sugano S (2004). Large-scale collection and characterization of promoters of human and mouse genes. In Silico Biol.

[B77] Wingender E (2004). TRANSFAC, TRANSPATH and CYTOMER as starting points for an ontology of regulatory networks. In Silico Biol.

[B78] Agresti A (1992). A survery of exact inference for contingency tables. Statistical Science.

[B79] Gentleman RC, Carey VJ, Bates DM, Bolstad B, Dettling M, Dudoit S, Ellis B, Gautier L, Ge Y, Gentry J, Hornik K, Hothorn T, Huber W, Iacus S, Irizarry R, Leisch F, Li C, Maechler M, Rossini AJ, Sawitzki G, Smith C, Smyth G, Tierney L, Yang JY, Zhang J (2004). Bioconductor: open software development for computational biology and bioinformatics. Genome Biol.

[B80] Li L, Carter J, Gao X, Whitehead J, Tourtellotte WG (2005). The neuroplasticity-associated arc gene is a direct transcriptional target of early growth response (Egr) transcription factors. Mol Cell Biol.

[B81] Tabuchi A, Sakaya H, Kisukeda T, Fushiki H, Tsuda M (2002). Involvement of an upstream stimulatory factor as well as cAMP-responsive element-binding protein in the activation of brain-derived neurotrophic factor gene promoter I. J Biol Chem.

[B82] Tao X, Finkbeiner S, Arnold DB, Shaywitz AJ, Greenberg ME (1998). Ca2+ influx regulates BDNF transcription by a CREB family transcription factor-dependent mechanism. Neuron.

[B83] Englander EW, Wilson SH (1990). Protein binding elements in the human beta-polymerase promoter. Nucleic Acids Res.

[B84] Ishidoh K, Suzuki K, Katunuma N, Kominami E (1991). Gene structures of rat cathepsins H and L. Biomed Biochim Acta.

[B85] Sheng M, Dougan ST, McFadden G, Greenberg ME (1988). Calcium and growth factor pathways of c-fos transcriptional activation require distinct upstream regulatory sequences. Mol Cell Biol.

[B86] Sassone-Corsi P, Visvader J, Ferland L, Mellon PL, Verma IM (1988). Induction of proto-oncogene fos transcription through the adenylate cyclase pathway: characterization of a cAMP-responsive element. Genes Dev.

[B87] Ehrengruber MU, Muhlebach SG, Sohrman S, Leutenegger CM, Lester HA, Davidson N (2000). Modulation of early growth response (EGR) transcription factor-dependent gene expression by using recombinant adenovirus. Gene.

[B88] Dean DC, Blakeley MS, Newby RF, Ghazal P, Hennighausen L, Bourgeois S (1989). Forskolin inducibility and tissue-specific expression of the fibronectin promoter. Mol Cell Biol.

[B89] James AB, Conway AM, Morris BJ (2006). Regulation of the neuronal proteasome by Zif268 (Egr1). J Neurosci.

[B90] Mayer RE, Khew-Goodall Y, Stone SR, Hemmings BA (1990). Expression and organization of protein phosphatase 2A catalytic subunit genes. Adv Second Messenger Phosphoprotein Res.

[B91] Allore RJ, Friend WC, O'Hanlon D, Neilson KM, Baumal R, Dunn RJ, Marks A (1990). Cloning and expression of the human S100 beta gene. J Biol Chem.

[B92] Rice DA, Aitken LD, Vandenbark GR, Mouw AR, Franklin A, Schimmer BP, Parker KL (1989). A cAMP-responsive element regulates expression of the mouse steroid 11 beta-hydroxylase gene. J Biol Chem.

[B93] James AB, Conway AM, Thiel G, Morris BJ (2004). Egr-1 modulation of synapsin I expression: permissive effect of forskolin via cAMP. Cell Signal.

[B94] Thiel G, Schoch S, Petersohn D (1994). Regulation of synapsin I gene expression by the zinc finger transcription factor zif268/egr-1. J Biol Chem.

[B95] Deutsch PJ, Hoeffler JP, Jameson JL, Lin JC, Habener JF (1988). Structural determinants for transcriptional activation by cAMP-responsive DNA elements. J Biol Chem.

