# Supplementary figures and images for: A comparative genomics approach to identifying the plasticity transcriptome
Source: BMC Neurosci. 2007 Mar 13;8:20. doi: 10.1186/1471-2202-8-20 (PMC1831778; doi:10.1186/1471-2202-8-20)

Predicted fraction of true positives

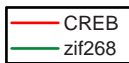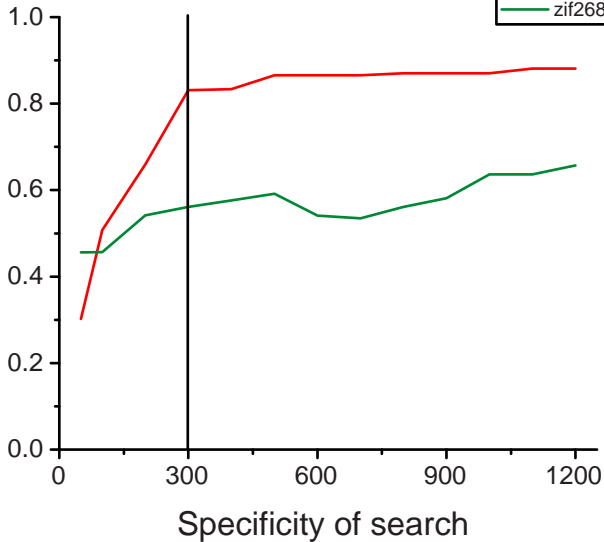

Supplement: Additional File 10 — Comparative genomics as a metric for transcription factor targets quality. Transcription factor binding site searches were done with varying correction scores that correspond to the specificity of the search. The log of the specificity is subtracted from every subsequence scored by the program to correct for sequence length (see Methods). A higher specificity means a smaller number of higher quality binding sites are used. The predicted fraction of true positives or positive predictive value is defined as (true positives)/(true positives + false positives). This measure is estimated as (observed sites - expected sites)/(observed sites). The observed sites are the targets verified by comparative genomics while the expected sites are the number of binding sites one would find by chance if comparing independent human/mouse datasets. [file 1471-2202-8-20-S10.pdf]
